# Linking a History of Childhood Abuse to Adult Health among Canadians: A Structural Equation Modelling Analysis

**DOI:** 10.3390/ijerph16111942

**Published:** 2019-05-31

**Authors:** Margherita Cameranesi, Lisa M. Lix, Caroline C. Piotrowski

**Affiliations:** 1 Applied Health Sciences PhD Program, Faculty of Graduate Studies, University of Manitoba, 311 Human Ecology Building, 35 Chancellors Cir, Winnipeg, MB R3T 2N2, Canada; 2 Department of Community Health Sciences, Max Rady College of Medicine, Rady Faculty of Health Sciences, University of Manitoba, S113-750 Bannatyne Avenue, Winnipeg, MB R3E 0W3, Canada; lisa.lix@umanitoba.ca; 3 Department of Community Health Sciences, Max Rady College of Medicine, Rady Faculty of Health Sciences, University of Manitoba, 217 Human Ecology Building, 35 Chancellors Cir, Winnipeg, MB R3T 2N2, Canada; caroline.piotrowski@umanitoba.ca

**Keywords:** adult survivor, family/domestic violence, child abuse, structural equation modelling, path analysis, life course health development

## Abstract

A history of childhood abuse has been linked to serious and long-lasting problems in adulthood. We developed two theoretical models concerning how early adverse experiences affect health in adulthood, and we tested the empirical fit of the two models in a population-based representative sample of Canadian adults (N = 25,113) using a structural equation modelling (SEM) technique, path analysis. The first model included direct pathways by which a history of three types of childhood abuse—exposure to intimate partner violence, physical abuse, and sexual abuse—affected adult physical and mental health, as well as indirect pathways by which perceived social support and everyday life stress acted as mediators of these associations. The second model included only indirect pathways and tested mediating effects. Global statistics indicated that both models were a good fit to the data, and local statistics supported the hypothesized associations between independent, dependent, and mediator variables.

## 1. Introduction

Child abuse can be defined as any form of physical, emotional/psychological, and/or sexual act of mistreatment or lack of care that results in emotional harm and/or physical injury to a child or youth under the age of 18 [1]. It includes all types of abuse, as well as the neglect of physical, emotional, and educational needs of the child, inadequate supervision, and the exposure to intimate partner violence (IPV). International studies reveal that one in four adults report having been physically abused as children, and one in five women and one in 13 men report having been sexually abused as a child [2]. Additionally, more than 35% of children worldwide are subject to emotional/psychological abuse and to neglect [3], which is the most common form of child maltreatment worldwide. In North America, more than 30% of children experience some form of abuse annually [4]. 

Abuse substantially affects the physical and mental health of children and youth and can also lead to negative long-lasting health outcomes in adulthood and later in life [4,5,6]. As reported in a recent Annual Research Review [7], the association between a history of childhood abuse and poor psychological and physical functioning in adulthood is well documented in both retrospective and prospective studies, showing that child physical, sexual, and emotional abuse is one of the most significant risk factors for poor physical and mental health in adulthood [8]. Survivors of childhood abuse show a higher prevalence of a wide range of mental health difficulties such as depression, anxiety, bipolar disorder, Post-Traumatic Stress Disorder (PTSD) symptoms, eating disorders, suicidal ideation, dissociation and dissociative disorders, substance use disorders, psychotic disorders, and personality disorders, compared to adults without such a history [5,9,10,11,12,13,14,15,16,17,18,19,20]. 

Childhood maltreatment is also a critical risk factor for later relationship-oriented problems, such as risky sexual behaviours, dating violence or IPV, narcissistic vulnerability, and relationship-related feelings of anxiety, guilt, fear, and shame, as well as antisocial outcomes such as trait anger, hostility, substance abuse, criminal behaviours, and physical aggression [21,22,23,24,25]. 

First investigated by Felitti and colleagues [26] a history of childhood abuse has been linked to a vast array of adult physical negative health outcomes such as negative perceptions of physical health, disability, cardiovascular diseases, obesity and diabetes, inflammation, metabolic syndrome, arthritis, cancer, gastrointestinal disorders, and pain disorders [27,28,29,30,31,32]. It is important to note that many of these studies relied upon non-probability sampling techniques, and that very few have investigated this phenomenon in a Canadian population. 

More recently, scholars have begun to investigate the factors that may mediate the association between a history of childhood abuse and later negative health outcomes, such as social support and sensitivity to stress [33,34,35,36]. There is growing evidence that people who experienced childhood abuse may be especially sensitive to stress [37,38], while social support can act as a buffer against the harmful effects of stressful events, such as childhood abuse [34]. However, different studies show mixed results regarding the specific role that social support and sensitivity to stress may play as mediators [10,39,40], making it a critical area for further exploration. 

### 1.1. Sex Differences

Findings concerning sex differences in the linkages between a history of childhood abuse and adult health are not consistent. While in some investigations sex has not been identified as a significant moderating factor in the relationship between abusive childhood experiences and adult physical and mental health [41,42,43], other studies have showed that sex interacts with the type of child abuse experienced to determine specific negative effects on adult physical and mental health [15,44,45,46,47,48]. Furthermore, the studies indicating differential effects of childhood abuse on the health of men and women show divergent results regarding the sex of those adults more at risk of developing physical and/or psychological symptoms following childhood abuse. These studies also show some differences concerning the specific physical and psychological health problems men and women with a history of child abuse exhibit during adulthood. 

Specifically, most of the research on sex differences in the development of mental health difficulties in adults with a history of childhood sexual abuse shows that women tend to be more negatively affected by this experience; compared to men with a history of sexual abuse during childhood, significantly more women who have been sexually abused during childhood develop at least one psychiatric disorder during adulthood [49], while they are significantly more vulnerable to suicide attempts than comparable men [50,51], and they show higher rates of major depressive disorders as well as alcohol consumption and cigarette smoking [45,47]. For instance, Verona and her colleagues [48] recruited an ethnically diverse sample of 304 adults and used structural equation modelling (SEM) to test a model of sex differences in the associations between childhood abuse and later drug use. Their findings revealed that childhood sexual and physical abuse were related to increased drug use in both sexes, but past abuse accounted for a larger part of women’s drug use compared to men’s, with different linkages between abuse types and drug use for men and women; for women, only childhood sexual abuse was positively associated with drug use, while for men only physical childhood abuse was positively associated with drug engagement. It is critical to emphasize that the sex differences found in some of the literature are strongly dependent on the measures used to assess childhood abuse and health, as well as on the population considered in the analysis. As mentioned, there are several studies that report no sex differences in the development of health problems following childhood abuse, while other studies report some differences, and these mixed results call for a deeper investigation of the issue. 

The present study is based upon the Life Course Health Development (LCHD) framework [52], which replicates and extends the literature on the linkages between childhood abuse and adult physical and mental health. Specifically, the purpose of the present research is threefold: (1) To investigate direct and indirect mechanisms by which childhood abuse experiences impact later adult physical and mental health in a population-based sample of Canadians using a SEM technique, path analysis; (2) to test if perceived social support and everyday life stress mediate the effects of childhood abuse on self-reported physical and mental health among Canadians; and (3) to examine sex differences in the effects of childhood abuse experiences on adult physical and mental health. The first step in conducting our investigation was the development of two nested competitive path models describing these associations using the LCHD framework.

### 1.2. Theoretical Models

The LCHD framework proposed by Halfon and colleagues [52] outlines how interactions between health determinants affect health outcomes across the life course and explains how health develops over an individual’s lifetime. The framework emphasizes the unique vulnerabilities of children that call for systematic attention to risk and protective factors early in life, recognizing that health is an emerging capacity of human beings that dynamically develops over time in response to multiple nested and ever changing genetic, biological, behavioural, social, and economic contexts [53]. According to this perspective, different adult health trajectories are the product of cumulative risk and protective factors and other influences that are programmed into bio-behavioural regulatory systems during critical and sensitive periods. 

**Figure 1:**
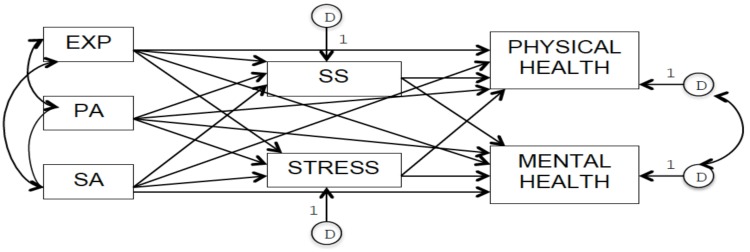
Model 1 representing direct and indirect effects of childhood exposure (EXP) to intimate partner violence (IPV), physical abuse (PA), and sexual abuse (SA) on adult physical and mental health through adult self-perceived social support (SS) and self-perceived everyday life stress (STRESS).

**Figure 2:**
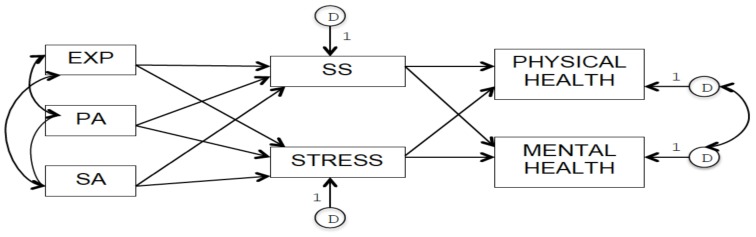
Model 2 representing indirect effects of childhood exposure to IPV (EXP), physical abuse (PA), and sexual abuse (SA) on adult physical and mental health mediated by adult self-perceived social support (SS) and self-perceived everyday life stress (STRESS).

The LCHD framework guided the development of our theoretical models that included direct and indirect associations between a history of childhood abuse and adult physical and mental health. We focused on three adverse childhood experiences (ACEs): Exposure to IPV, physical abuse, and sexual abuse. We decided to focus specifically on these three ACEs because they are all measured in the 2012 Canadian Community Health Survey (CCHS)–Mental Health [54], which represents our data source. In Model 1 (see Figure 1), all three forms of abuse (exogenous variables) were hypothesized to act as risk factors directly and negatively impacting self-rated adult physical and mental health (endogenous variables). Furthermore, following from the LCHD framework, we hypothesized that these three forms of abuse in childhood increase later life perception of everyday stress and decrease perceived social support (endogenous variables and mediators) through the modification of bio-behavioural systems, therefore negatively impacting physical and mental health in adulthood also through these indirect pathways. In order to test this mediational model, in Model 2 (see Figure 2), we included only the indirect effects of these three types of childhood abuse on adult physical and mental health through self-perceived social support and self-perceived everyday life stress in adulthood. In both models, we hypothesized the presence of positive correlations between the three forms of childhood abuse due to shared risk factors, and between common determinants of adult physical and mental health not included in our models, such as socio-economic status [55,56,57].

## 2. Materials and Methods

### 2.1. Study Design

#### Data Source

To test the empirical fit of the theoretical models, we performed a secondary analysis of quantitative data gathered from the Public Use Microdata File of the 2012 Canadian Community Health Survey (CCHS)–Mental Health [58]. The CCHS–Mental Health is an ongoing biannual national-level survey with a repeated cross-sectional design developed and administered by Statistics Canada [59]. The CCHS–Mental Health collects information about the factors, influences, and processes that contribute to mental health through a multidisciplinary approach focusing on health, social, and economic determinants. Specifically, in addition to questions assessing adult self-rated physical and mental health, and perceived stress and social support, the 2012 CCHS–Mental Health included a set of questions designed to investigate three types of child abuse; namely, exposure to IPV, physical abuse, and sexual abuse. 

### 2.2. Study Population

The target population of the 2012 CCHS–Mental Health survey was all persons 15 years of age and over living in the ten Canadian provinces, excluding residents of the Yukon, Northwest Territories, and Nunavut, persons living on reserves or other Aboriginal settlements, full-time members of the Canadian Forces, and full-time residents of institutions [58]. Altogether, these exclusions represented less than 3% of the target population. Since the questions assessing child abuse were only administered to respondents aged 18 years and older, the study sample included all participants to the 2012 CCHS–Mental Health that were 18 years of age and older. 

As in all Statistics Canada surveys, the sampling technique used in the 2012 CCHS–Mental Health followed a multi-stage probability sampling procedure. Specifically, a three-stage design was used to select the respondents for the 2012 CCHS–Mental Health (see [58] for a detailed description). Data were collected between January and December 2012 through computer assisting telephone interviewing (CATI) performed in the language of choice of the respondents (either English or French), and at the end of data collection a national response rate of 68.9% was achieved. 

### 2.3. Study Measures

#### 2.3.1. Child Abuse

Exposure to IPV during childhood was assessed with one question asking how many times the respondents before age 16 saw or heard a parent hit another adult in their home. Three questions evaluated physical abuse that occurred before age 16, assessing the number of times respondents were (1) slapped, hit, or spanked; (2) pushed, grabbed, shoved, or had things thrown at them; and (3) physically attacked. Two questions were used to assess child sexual abuse asking the number of times respondents before age 16 were (1) forced in unwanted sexual activities; and (2) forced to sexual touching. Whether or not respondents experienced non-contact sexual abuse was not assessed in the 2012 CCHS–Mental Health, and therefore in the present study we were not able to investigate the differential effects of contact and non-contact childhood sexual abuse on adult health. Respondents rated the frequency of each item on a 5-point scale, from never to more than 10 times. Since according to the LCHD framework the sole presence of any type of child abuse works as a risk factor, and because our sample was heterogeneous in age and we had no data on the length of the abuse, for the purpose of the present analysis we created three binary variables indicating the presence or absence of the three types of child abuse.

#### 2.3.2. Social Support

Social support was assessed using a short version of the 24-item Social Provisions Scale (SPS) developed by Cutrona and Russell [60] to evaluate the degree to which respondents’ social relationships provided various dimensions of social support. The shortened SPS-10 included 10 items positively worded, two per each of the five subscales retained out of the six included in the original version. The items aimed to measure the availability of social support through statements such as “I have someone to talk to about decisions in my life” or “I have close relationships that make me feel good” and were rated on a 4-point scale, from strongly agree to strongly disagree. The scale was validated on a nationally representative sample of 2433 people recruited in the southwest region of Montreal [61] and showed strong concurrent validity with the original scale (r = 0.930). The Cronbach’s alpha for the global scale was 0.880; in our analyses, we used the total score of the SPS-10 ranging from 10 to 40, indicating low and high social support, respectively. 

##### Stress

Perceived life stress was assessed with a single question asking respondents to rate the amount of stress they perceived in their everyday lives on a 5-point scale, from not at all stressful (1) to extremely stressful (5). 

##### Physical Health

Self-rated physical health was assessed using one question asking respondents to rate their physical health on a 5-point scale, from excellent (5) to poor (1). 

##### Mental Health

Self-rated mental health was assessed using one question asking respondents to rate their mental health on a 5-point scale, from excellent (5) to poor (1). 

##### Sociodemographic Characteristics

A series of questions was used to gather information regarding respondents’ sex, age, marital status, occupation, level of education, and income. 

### 2.4. Data Analysis

All statistical analyses were performed using the software SAS 9.4 (SAS Institute Inc., Cary, NC, USA) and the SEM path (SAS Institute Inc., Cary, NC, USA); analyses were executed using PROC CALIS. Prior to conducting path analysis, descriptive statistics were generated, and distributional assumptions and patterns of missing data were assessed. No outliers were identified. Descriptive statistics were weighted according to Statistics Canada guidelines [58] to ensure that the data were representative of the Canadian population. As we expected due to the sensitive nature of the items, the variables with the highest proportion of missing data were those evaluating child abuse, with approximately 9.5% of missing data per variable. However, this represents an overestimation of the portion of missing data because they also included all participants younger than 18 who were not asked the questions on child abuse. The remaining proportions of missing data did not reach 3% (highest: 2.9% for social support). Profile analyses were performed to assess whether Canadians with missing data in the questions on child abuse were significantly different from those who provided answers, with respect to sociodemographic characteristics of sex, age, and occupation. Since, as expected, they differed significant only with regard to their age, and considering the relatively low proportion of missing data, we decided to exclude all cases of missing data from our analyses. The variables “perceived social support” and “everyday stress in adulthood” displayed the largest skewness (−1.3) and kurtosis (6.2), respectively. Collinearity assessment was also performed computing bivariate correlations of all study variables; all variables were significantly correlated at the bivariate level, and no collinearity problems were detected (−0.11 < *r* < 0.69). 

Following the recommendations of Kline [62], since our models included ordinal categorical outcome variables and because of a violation of the normality assumptions, we adopted the diagonally weighted least squares (DWLS) estimation method. Unfortunately, PROC CALIS in SAS with DWLS does not provide the standard errors and p levels of the parameter estimates. However, recent studies have shown that standard errors are likely to be biased under many data-analytic conditions [63] and, therefore, assessments of statistical significance of parameter estimates may not be accurate. For these reasons, in this investigation, we presented the parameter estimates of direct effects and covariances by providing the standardized and unstandardized coefficient only. Following this, we conducted global fit and local fit testing using the indices described below.

DWLS performs well when the sample size is above N = 200 and when categorical indicators are not markedly skewed; therefore, it was a suitable estimation method for our analyses. Global model fit was assessed using: 1) The Standardized Root Mean Square Residual (SRMR), 2) the Goodness-of-Fit Index and the Adjusted Goodness-of-Fit Index (GFI and AGFI), and 3) the Bentler–Bonett Normed Fit Index (NFI) [64]. SRMR, GFI, and AGFI are absolute measures of fit that indicate the degree to which the hypothesized model reproduces the sample data, while NFI is an incremental fit index that measures the proportional improvement in fit when the hypothesized model is compared with a restricted, nested baseline model [65]. RMR decreases as goodness of fit increases and is bounded below by zero, while GFI, AGFI, and NFI increase as goodness of fit increases and are bounded above by 1.00. As recommended by several scholars [62,66], when analyzing categorical data values of SRMR ≤ 0.08, GFI, AGFI, and NFI ≥ 0.95 indicate a good global model fit. Following the global model fit analyses, we conducted local fit testing examining unstandardized and standardized parameter estimates and coefficients of determinations (R^2^). The indirect causal effects of the three types of childhood abuse on adult physical and mental health were computed by multiplying the direct effects of childhood exposure to IPV, physical abuse, and sexual abuse on the mediators, social support and stress, and the direct effects of the mediators on the endogenous variables, physical and mental health. In SAS, PROC CALIS with DWLS does not provide the model chi-square and the Steiger–Lind Root Mean Square Error of Approximation (RMSEA).

## 3. Results

### 3.1. Descriptive Statistics

In 2012, 25,113 Canadians participated in the CCHS–Mental Health survey and were representative of a population of 28,314,716. Table 1 reports the sociodemographic characteristics of the total sample from which the subsample of respondents aged 18 years and older used in the present study was drawn (N = 21,958). 

In the FUMF, respondents’ age is categorized into three age groups (see Table 1), with the youngest group including all those aged 15 to 34 years. Therefore, we did not have access to the age of each participant individually and were not able to distinguish between those who did not answer the questions on child abuse from those who were not asked these questions, with a consequent overestimation of the missing data. Study participants were almost evenly divided between the sexes and between age groups, with the greatest proportion of respondents aged from 35 to 54 years (N = 8,865; 35.3%). Almost half the sample reported being married, and approximately 63% of participants were employed at the time of the survey. Nearly 14% of the sample reported exposure to IPV before age 16, while 7% and 5% of respondents reported childhood physical and sexual abuse, respectively. Overall, Canadians positively self-evaluated their general and mental health, the distributions of which were both skewed to the left with a median of 3 (3 = Good), while perceiving their lives only a bit stressful (median = 3). The mean of social support was 36 with a standard deviation of 14.

### 3.2. Model Fit Testing in the Total Sample

**Global fit testing.** After deleting the observations with missing values and those of Canadians younger than 18 years, we performed the path analyses on the covariance matrix of N = 21,958 cases. Both models were identified, with 28 observations and 27 parameters to estimate (df = 1) in Model 1, and 28 observations and 21 parameters to estimate in Model 2 (df = 7), representing the most parsimonious model. Both models showed a good fit to the data (Model 1, SRMS = 0.015, GFI = 0.998, AGFI = 0.95, and NFI = 0.99; Model 2, SRMS = 0.03, GFI = 0.993, AGFI = 0.97, and NFI = 0.96). 

**Local fit testing.** The path models we tested included both direct and indirect effects of child abuse on adult physical and mental health. Table 2 reports the unstandardized and standardized parameter estimates for all path estimations of the direct effects included in the two models, as well as the covariance estimates. 

As expected, in Model 1, the three types of childhood abuse had direct negative effects on adult self-rated physical and mental health. In both models, the three types of childhood abuse experiences had negative effects on perceived adult social support and a positive effect on perceived everyday life stress. Childhood abuse in the form of exposure to IPV, physical abuse, and sexual abuse was associated with a decrease in self-reported physical and mental health, as well as a decrease in perceived adult social support and an increase in perceived everyday life stress. Furthermore, in both models, adult perceived social support was positively related to self-rated physical and mental health, while adult perceived everyday life stress was negatively associated with self-rated physical and mental health: The perception of higher levels of social support was related to higher self-rated physical and mental health, while the perception of higher level of everyday stress was associated with a decrease in self-rated physical and mental health. The parameter estimates in Model 2 are larger in absolute values than those in Model 1, especially for the estimation of the pathways connecting the three types of childhood abuse to adult perceived social support and perceived everyday life stress. As shown in Table 3, childhood exposure to IPV, physical abuse, and sexual abuse affected adult physical and mental health through two indirect pathways mediated by adult perceived social support and everyday life stress, respectively.

Model 1 explained 8.9% and 19.4% of the variability in self-rated adult physical health and mental health, respectively, as well as 2.3% and 1.6% of the variability in perceived everyday life stress and social support, respectively. Model 2 explained 9.4% and 20.7% of the variability in self-rated adult physical health and mental health, respectively, as well as 3.4% and 2.6% of the variability in perceived everyday life stress and social support, respectively. Therefore, compared to Model 1, Model 2 accounted for a greater portion of variability in mediators and outcome variables. According to the LCHD framework, the small values of R^2^ we obtained can be due to the many risk and protective factors not examined here that, in addition to childhood abuse, affect both physical and mental health over time, such as sex, age, and socio-economic status.

In order to test the robustness of our findings, we conducted a sensitivity analysis testing global and local model fit of both models using three additional methods of estimation: Maximum likelihood (ML), full information maximum likelihood (FIML), and weighted least squares (WLS). As reported in the Appendix, the results we obtained were comparable across the three estimation methods and very similar to those obtained using DWLS, strengthening our confidence in the good empirical fit of our theoretical models and in the accuracy of the parameter estimates. 

The results of global fit testing and local fit testing showed that both models were a good fit to the data. However, compared to Model 1, Model 2 that included only indirect effects represents a more parsimonious model, and because its parameter estimates and coefficients of determination are larger in absolute value, it represents the best model to explain the associations between childhood abuse and adult poor physical and mental health. Therefore, we further tested the validity of the mediational model (Model 2) in two subsamples of Canadian men and women. 

### 3.3. Multiple-Group Analysis

In the final step of our SEM analysis, multigroup analyses were conducted to examine whether our best model was invariant across sexes. We tested the empirical fit of Model 2 in two subsamples of Canadians stratified by sex: 1) A sample of males (n_m_ = 11,340); and 2) a sample of females (n_f_ = 13,773), to assess the validity of our preferred model in describing the indirect associations between childhood abuse and adult physical and mental health among Canadian men and women. As per the SEM analyses performed on the total sample, the model showed a good fit for both subsamples of males and females (see Table 4). For the sample of males, SRMR = 0.02, GFI = 0.99, AGFI = 0.98, and NFI = 0.97, while for the female sample, SRMR = 0.035, GFI = 0.99, AGFI = 0.97, and NFI = 0.96. 

The parameter estimates of the two analyses stratified by sex were identical in sign and very similar in magnitude, with most of the estimates being very similar if not identical for the two subgroups. As expected based on previous research on childhood sexual abuse [50,51], the greatest differences were identified in the strength of the negative associations between childhood sexual abuse and adult perceived everyday stress, and between adult perceived life stress and self-rated physical and mental health in adulthood, which were both stronger in the female subsample. 

## 4. Discussion

The present investigation filled some critical gaps in the literature by using a Life Course Health Development (LCHD) approach to empirically test how early risk factors in childhood affect health in adulthood in a nationally representative sample of Canadians. Our main purpose was to test the potential mediating role that adult perceived social support and everyday life stress may play in explaining the associations between childhood abuse and adult poor physical and mental health, in order to gain a better understanding of the specific mechanisms responsible for the long lasting effects of childhood abuse on adult health. As hypothesized in our theoretical models, childhood exposure to IPV, physical abuse in childhood, and sexual abuse in childhood were directly and negatively related to adult self-rated physical and mental health, as well as negatively associated with adult self-perceived social support and positively associated with adult perceived everyday life stress. These findings provide support for other work that found that perceived life stress and social support were associated to self-rated physical and mental health among adult Canadians [34,38]. It is important to highlight that a history of any type of childhood abuse increased the level of everyday life stress perceived by adult Canadians while simultaneously decreasing their perception of availability of social support. In turn, the perception of being under high everyday life stress in adulthood was associated with poorer self-rated physical and mental health, while the perception of high availability of social support as an adult was associated with higher self-rated physical and mental health. Therefore, everyday life stress and social support mediated the effects of childhood abuse on adult physical and mental health. Furthermore, our findings were robust across different methods of estimations and across analyses stratified by sex, providing further evidence that this model accurately describes the linkages between childhood abuse and adult physical and mental health among Canadians. 

Our findings support a body of work that shows that social support could function as a buffer against the development of psychopathological and physical symptoms in individuals exposed to early trauma and that one of the possible mechanisms through which such symptoms can arise is the stress system [10,39]. However, due to the small amount of variance explained in both our models and the associated limited clinical value of our findings, the results of our study should be interpreted with caution, and additional investigations should be conducted to replicate these findings. The identification of the specific mechanisms that partially or entirely account for the onset of physical and psychological pathology in adult survivors of childhood abuse represents a critical step towards planning and implementing more effective intervention programs aimed to reduce the negative health outcomes associated with early life abuse and adverse childhood experiences. Toward this end, research that applies the SEM technique can be a very valuable resource that scholars investigating the long-lasting effects of childhood abuse on adult health can use to test different direct and indirect causal models with the purpose of enhancing our understanding of the mechanisms that affect physical and mental health in this population. 

The results of path analysis stratified by sex depicted an interesting picture. While the model we developed appears to accurately describe the linkages between childhood abuse experiences and adult health for both men and women, some interesting minor differences were identified. In line with findings of other research conducted with US and European samples [47,48], in our sample of Canadian adults, childhood sexual abuse and adult perceived everyday life stress appeared to have a somewhat greater impact on women’s physical and mental health, compared to men. A history of childhood sexual abuse had a stronger negative impact on Canadian women’s perception of everyday life stress than for men with such a history, increasing the amount of stress that these women perceived every day. Furthermore, perceiving high levels of everyday life stress had a more negative impact on the physical and mental health of Canadian women with a history of childhood abuse than on men who were abused during childhood. We believe that recent research findings on enduring neurobiological effects of childhood abuse and sex-dependent differences in brain morphology of adult men and women who were abused during childhood [7,67,68] can help to explain our findings on the slightly different impact that different types of childhood abuse have on the health of Canadian men and women.

Our study has some limitations. Beyond the scope of the current study, our models did not include additional variables that represent important determinants of health such as socioeconomic status, level of education, and employment status. The use of cross-sectional data precludes making causal conclusions due to the limitations of retrospective data. Although we used a large population-based sample, our findings should not be interpreted as representing the experience of all Canadians. Future research should include other social determinants of health, including family and community risk and protective factors, to broaden our understanding of the effects of childhood abuse on adult physical and mental health. Replications of the present analyses in independent samples are also needed to assess the generalizability of our findings. An additional shortcoming of our study is that the different forms of child abuse were assessed using a very small number of questions, with IPV exposure being measured using only a single question. This could have affected our findings in two ways. First, beliefs regarding what types of behaviour (or lack thereof) and parenting constitute child abuse and neglect may have varied across study participants, and the use of multiple items to assess each form of child abuse would have provided a more valid assessment of these variables. Second, the literature has largely shown that the more questions are used in eliciting child abuse victimization, the higher the number of child abuse episodes that participants report is [69]. Therefore, the adults who participated in the 2012 CCHS–Mental Health (see [38]) may have underreported their childhood abuse experiences.

## 5. Conclusions

In this study, we tested the empirical fit of two models describing the linkages between early childhood abuse experiences and later adult physical and mental health in a population-based representative sample of Canadian adults (N = 25,113) using the structural equation modelling (SEM) technique path analysis. The first model included direct pathways by which a history of three types of childhood abuse—exposure to IPV, physical abuse, and sexual abuse—affected adult physical and mental health, as well as indirect pathways by which perceived social support and everyday life stress acted as mediators of these associations. The second model included only indirect pathways and tested mediating effects. 

Our main purpose was to test the potential mediating role that adult perceived social support and everyday life stress may play in explaining the associations between childhood abuse and adult poor physical and mental health, in order to gain a better understanding of the specific mechanisms responsible for the long lasting effects of childhood abuse on adult health. As hypothesized in our theoretical models, childhood exposure to IPV, physical abuse in childhood, and sexual abuse in childhood were directly and negatively related to adult self-rated physical and mental health, as well as negatively associated with adult self-perceived social support and positively associated with adult perceived everyday life stress. These findings supported previous research in this field and should be replicated using primary population-based longitudinal data. 

## Figures and Tables

**Table 1:** Sociodemographic characteristics of the total sample (N = 25,113).

Variable	N (%)	Variable	N (%)
SEX Male Female	12,381 (49.3) 12,732 (50.7)	OCCUPATION Employed Unemployed	15,822 (63.1) 8789 (34.9)
AGE GROUP		Unable to work	502 (2.0)
15–34 35–54 55–74 ≥75	7936 (31.6) 8865 (35.3) 6580 (26.2) 1732 (6.9)	PERSONAL INCOME <$20,000 $20,000–$49,999 ≥$50,000	8238 (32.8) 9743 (38.8) 7132 (28.4)
MARITAL STATUS Married Common-law Widowed Divorced/Separated Single	12,381 (49.3) 2712 (10.8) 1230 (4.9) 2009 (8.0) 6781 (27)	HOUSEHOLD INCOME <$20,000 $20,000–$79,999 ≥$80,000	1155 (4.6) 12,080 (48.1) 11,878 (47.3)

Note: Unweighted sample, N = 25,113; Population, N = 28,314,716.

**Table 2:** Parameter estimates of direct effects and covariances.

Parameter	Model 1		Model 2	
	Unst.	Stand.	Unst.	Stand.
EXP → PHYSIC_H EXP → MENTAL_H	−0.04 −0.09	−0.01 −0.03	- -	- -
PA → PHYSIC_H PA → MENTAL_H SA → PHYSIC_H SA → MENTAL_H	−0.14 −0.15 −0.24 −0.25	−0.04 −0.04 −0.06 −0.07	- - - -	- - - -
EXP → SS EXP → STRESS PA → SS PA → STRESS SA → SS SA → STRESS	−0.71 0.21 −1.16 0.19 −0.68 0.33	−0.06 0.08 −0.07 0.05 −0.04 0.08	−0.82 0.24 −1.44 0.24 −1.13 0.41	−0.07 0.08 −0.09 0.06 −0.06 0.10
SS → PHISIC_H SS → MENTAL_H STRESS → PHYSIC_H STRESS → MENTAL_H	0.05 0.06 −0.17 −0.27	0.21 0.28 −0.17 −0.29	0.05 0.06 −0.19 −0.30	0.23 0.30 −0.19 −0.33
CV EXPPA CV EXPSA CV PASA CVDPHYSIC_HDMENTAL_H	0.04 0.02 0.02 0.32	0.39 0.23 0.28 0.34	0.04 0.02 0.02 0.31	0.39 0.23 0.28 0.33

Note: EXP, exposure to IPV; PV, physical abuse; SA, sexual abuse; SS, social support; STRESS, everyday stress; PHYSIC_H, physical health; MENTAL_H, mental health; CV, covariance; D, disturbances. Unst., unstandardized; Stand., standardized.

**Table 3:** Parameter estimates of indirect effects.

Indirect Path	Estimations	
	Model 1	Model 2
EXP → SS → PHYSIC_H	−0.715 (0.05) = −0.03	−0.82 (0.05) = −0.04
PA → SS → PHYSIC_H	−1.16 (0.05) = −0.06	−1.44 (0.05) = −0.07
SA → SS → PHYSIC_H	−0.68 (0.05) = −0.03	−1.13 (0.05) = −0.06
EXP → SS → MENTAL_H	−0.715 (0.06) = −0.04	−0.82 (0.065) = −0.05
PA → SS → MENTAL_H	−1.16 (0.06) = −0.07	−1.44 (0.065) = −0.09
SA → SS → MENTAL_H	−0.68 (0.06) = −0.04	−1.13 (0.065) = −0.07
EXP → STRESS → PHYSIC_H	0.215 (−0.17) = 0.04	0.24 (−0.19) = −0.04
PA → STRESS → PHYSIC_H	0.19 (−0.17) = −0.03	0.24 (−0.19) = −0.04
SA → STRESS → PHYSIC_H	0.33 (−0.17) = −0.06	0.41 (−0.19) = −0.08
EXP → STRESS → MENTAL_H	0.215 (−0.27) = −0.06	0.24 (−0.30) = −0.07
PA → STRESS → MENTAL_H	0.19 (−0.27) = −0.05	0.24 (−0.30) = −0.07
SA → STRESS → MENTAL_H	0.33 (−0.27) = −0.09	0.41 (−0.30) = −0.12

Note: EXP, exposure to IPV; PV, physical abuse; SA, sexual abuse; SS, social support; STRESS, everyday stress; PHYSIC_H, physical health; MENTAL_H, mental health.

**Table 4:** Global fit indexes of Model 2 for two subgroups of Canadian males and females.

Fit Index	Male Subsample (n_m_ = 11,340)	Female Subsample (n_f_ = 13,773)
SRMR	0.02	0.03
GFI–AGFI	0.99–0.98	0.99–0.97
NFI	0.97	0.96

Note: SRMR, Standardized Root Mean Square Residual; GFI and AGFI, Goodness-of-Fit Index and Adjusted Goodness-of-Fit Index; NFI, Bentler–Bonett Normed Fit Index.
